# Non-ideal behavior of a treadmill depends on gait phase, speed, and weight

**DOI:** 10.1038/s41598-019-49272-0

**Published:** 2019-09-04

**Authors:** Austin Tielke, Jooeun Ahn, Hyunglae Lee

**Affiliations:** 1 School of Biological and Health Systems Engineering, Arizona State University, Tempe, AZ 85287 USA; 2 Department of Physical Education, Seoul National University, Seoul, 08826 Republic of Korea; 3 Institute of Sport Science, Seoul National University, Seoul, 08826 Republic of Korea; 4 School for Engineering of Matter, Transport, and Energy, Arizona State University, Tempe, AZ 85287 USA

**Keywords:** Biomedical engineering, Mechanical engineering

## Abstract

Noticeable differences exist between treadmill and overground walking; kinematics, kinetics, and muscle activation patterns differ between the two. Many previous studies have attributed the differences to changes in visual information, air resistance, and psychological effects such as fear. In this study, we demonstrate that no treadmill serves as an inertial frame of reference. Considering the linear momentum principle, the finite sampling rate of the controller, and the limited power of the treadmill motor, we predict that 1) the error of the treadmill speed periodically varies depending on the locomotion phase and 2) this non-ideal behavior becomes more evident as the locomotion speed or the weight of the walker increases. Experimental observation confirmed our predictions by quantifying the variation of the actual treadmill belt speed and the ground reaction force in the anterior–posterior direction for different locomotion speeds and subject weights. These results emphasize a need for design criteria like the minimum sampling rate and the minimum motor power that treadmill locomotion studies should consider.

## Introduction

Treadmills have been widely used for locomotion studies by virtue of their significant advantages: the speed can be controlled and the experiment can be done in a laboratory space, enabling simultaneous use of other stationary equipment. However, those who have walked or run on a treadmill for a sufficient period of time recognize the difference between treadmill and overground locomotion. Considering that the eventual goal of most locomotion studies is to provide better understanding or assistance to our daily, overground walking, the noticeable difference between treadmill and overground locomotion raises important questions about the validity of applying the treadmill study results to overground activities. A number of kinesiology and biomechanics studies have addressed this critical issue by quantifying differences in various aspects, including temporal gait parameters, kinematics, kinetics, muscle activation, and stability^1–10^.

Though detectable differences between treadmill and overground locomotion in various measures have been reported, understanding of the sources of the difference is limited. The main sources proposed by previous studies include the difference in air resistance, visual information, and psychological effects, including fear^10,11^. Most treadmill studies are based on the assumption that the coordinate system attached to the treadmill belt is close to an inertial frame of reference and observed differences emerge from causes other than the non-ideal behavior of a treadmill^11^. However, the linear momentum principle and the basic dynamics of feedback control system clearly inform us that no treadmill can serve as an inertial frame of reference. The runner or walker exerts significant time-varying force on the treadmill belt; the power of the electrical motor is limited; and the controller has a finite sampling rate, hence a finite time delay exists in the control loop. Consequently, the treadmill belt can be neither an ideal “flow source,” nor an inertial frame of reference.

A few studies have quantitatively addressed treadmill belt speed variation and its effect on locomotion. Savelberg *et al*. investigated the intra-stride belt speed variation and its effect on locomotion^12^. They concluded that the belt speed variations of treadmills affect the locomotion patterns significantly, and the variations depend on the power of the treadmill and the weight of the subject. Interestingly, Savelberg *et al*. reported that the speed of locomotion does not contribute to the belt speed variation. This important study suggested that the energy exchange between the subject and the treadmill plausibly causes the kinematic differences between overground and treadmill locomotion. Unfortunately, the force between the subject and the treadmill belt could not be measured directly because the instrumented treadmill was not yet devised when the study was conducted. Instead, the ground reaction force obtained from overground locomotion was used to estimate the energy exchange. More recently, Sloot *et al*. directly estimated the mechanical energy exchange between a subject and a treadmill by measuring the belt speed deviation and the ground reaction force using an instrumented treadmill^13^. They reported that the total energy exchange is less than 1.6% of the work performed on the center of mass, although the deviation of the belt speed is over 3%. While these studies quantified the inevitable but previously uninvestigated mechanical cause of differences between treadmill and overground locomotion, the underlying mechanics of the speed variation have not yet been quantitatively addressed. Savelberg *et al*. tried to model the relation between belt speed variation and other mechanical parameters, such as the weight of the subject and the power of the treadmill^12^. However, the model was obtained by multiple linear regression rather than mechanics; linear relations between the belt speed variation and all inputs are assumed and the corresponding coefficients were estimated by curve fittings.

In this study, we demonstrate that the error of the treadmill speed depends on gait phase, the weight of a subject, and locomotion speed. The mechanics of simple walking models provided an insight into the relation between the external perturbation to the treadmill belt and basic parameters: speed and weight. In our experiment, we directly measured the ground reaction force and the treadmill belt speed. The results verified the model prediction and showed that the speed variation clearly depended on the speed of locomotion as well as the weight of a subject and gait phase. This finding differs from the results of the previous study by Savelberg *et al*.^12^ but is consistent with the prediction based on mechanics.

## Predictions Based on Mechanics

A treadmill belt is typically driven by an electrical motor, and the belt speed is regulated by a controller. However, the power of the motor and the sampling rate are not infinite. Consequently, by Newton’s law, the force that the runner or walker applies to the belt causes acceleration or deceleration of the belt, at least until the next update of the control loop. In human locomotion, the ground reaction force varies periodically depending on the locomotion cycle. Therefore, the inevitable acceleration or deceleration of the treadmill belt (i.e., the error of the treadmill speed) should depend on the gait phase. In particular, the large ground reaction force around heel-strike (HS) and ankle push-off just before toe-off (TO) suggests that the speed variation of a treadmill belt will be more evident around those phases.

To understand the mechanical parameters that affect the treadmill belt speed, we used two simplified walking models: one presented by Ahn and Hogan^14,15^, and the other presented by Geyer, Seyfarth, and Blickhan^16^. The first model has rigid legs, whereas the second one has compliant springy legs. In both models, a point mass moves in a vertical plane under the influence of gravity. All numerical simulations and analyses were implemented in Matlab (Mathworks Inc., Natick, MA, USA). Numerical integration by the Runge-Kutta method was performed with a fixed step size of 10^−6^. The validity of the numerical simulation was checked by repeating simulations with a tenfold smaller step size.

### A walking model with rigid legs

**Figure 1:**
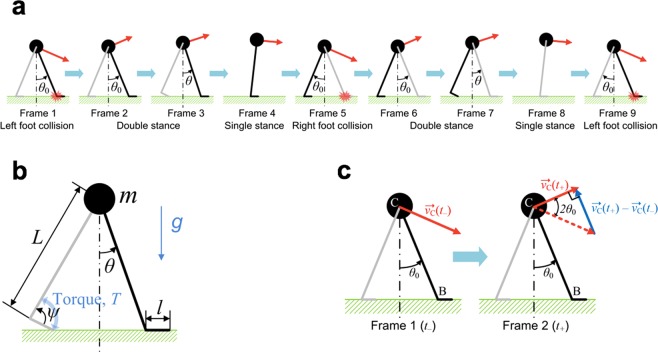
The simple walking model with ankle actuation. (**a**) One stride of the walking model^14^. The end and beginning of a step are the moments when the leading foot collides with the ground (Frames 1, 5, and 9). During double stance, the model moves as four linked bars (Frames 2, 3, 6, and 7). During single stance, the model moves as an inverted pendulum (Frames 4 and 8). (**b**) A schematic of the walking model^14^. A point mass is restrained by rigid massless legs. The trailing ankle is actuated whereas the hip joint and the leading ankle do not exert torque. (**c**) The foot–ground collision of the walking model. The leading heel, B, is landing on the ground. Frame 1 shows the snapshot at *t*_−_, the moment just before the heel-strike (HS), and Frame 2 shows the snap shot at *t*_+_, the moment right after HS. The geometry determines that the velocity of the point mass C changes its direction by 2*θ*_0_ due to the collision, and the angular momentum principle determines that the ratio of the speed right after the collision to the speed just before the collision should be cos(2*θ*_0_). Consequently, the difference between the linear momentum at *t*_+_ and the linear momentum at *t*_−_, or the impact due to the ground reaction force, should be parallel to the leading leg, or the line from B to C. (The blue vector at Frame 2 is parallel to line BC).

A schematic of the model defining its variables and sequential configurations during one stride are shown in Fig. 1. The swing leg can be moved instantaneously in front of the mass; scuffing is ignored. Each leg has a hip and an ankle. Ankle actuation provides propulsion whereas the hip joint is assumed to be a frictionless pivot, which cannot apply any torque. However, the angle between the legs is always reset as 2*θ*_0_ at the beginning of a step. Due to the assumption of massless legs, resetting the angle between the legs does not consume any energy. The ankle torque during double stance is determined as

1 $$T=k(\mu -\psi ),$$

where *T* is the plantar ankle torque at the trailing ankle, *ψ* is the ankle angle that is positive towards plantar flexion, and *μ* is the maximal plantar flexion angle.

The configuration of the model at the foot–ground contact is explicitly shown in Fig. 1c. At the collision of the leading foot (Frame 1), the velocity of the point mass changes instantaneously, while the direction changes by 2*θ*_0_ (Frame 2). By the angular momentum principle about the heel of the leading leg *B*,

2 $$\frac{d}{dt}\overrightarrow{{H}_{B}}+\overrightarrow{{v}_{B}}\times \overrightarrow{P}=\overrightarrow{{M}_{B}}=\overrightarrow{{r}_{BC}}\times m\overrightarrow{g},$$

where $\overrightarrow{{H}_{B}}$, $\overrightarrow{{v}_{B}}$, $\overrightarrow{P}$, $\overrightarrow{{M}_{B}}$, and $\overrightarrow{{r}_{BC}}$ denote the angular momentum about *B*, the velocity of point *B*, the linear momentum of the model, the external torque about *B*, and the position vector pointing *C* from *B*, respectively. The model has Frame 1 and Frame 2 of Fig. 1c occurring at *t*_−_ and *t*_+_ respectively; the collision between the foot and the ground occurs during the infinitesimal time interval between *t*_−_ and *t*_+_. Because the velocity of point *B* is zero, the second term on the left-hand side of Eq. (2) vanishes, and

3 $$\overrightarrow{{H}_{B}}({t}_{+})-\overrightarrow{{H}_{B}}({t}_{-})={\int }_{{t}_{-}}^{{t}_{+}}(\overrightarrow{{r}_{BC}}\times m\overrightarrow{g})dt.$$

The right-hand side of Eq. (3) equals zero because the time gap between *t*_−_ and *t*_+_ is infinitesimal, and the integrated term is not impulsive. Therefore, the angular momentum about *B* is conserved during the collision, and

4 $$\overrightarrow{{H}_{B}}({t}_{+})=\overrightarrow{{H}_{B}}({t}_{-}).$$

Though this conservation is a result of the dynamics of a highly simplified walking model, it is noteworthy that angular momentum is approximately conserved during actual human locomotion as well^17^. Consequently, to maintain angular momentum with the direction of the velocity changed by 2*θ*_0_, the magnitude of the velocity decreases, and the ratio of the speed of the mass right after the collision to the speed just before the collision becomes cos(2*θ*_0_);

5 $$|\overrightarrow{{v}_{C}}({t}_{+})|=\,\cos (2{\theta }_{0})|\overrightarrow{{v}_{C}}({t}_{-})|.$$

The ground reaction force generates impulse and changes the linear momentum of the model. The change of the momentum has the magnitude of $m|\overrightarrow{{v}_{C}}({t}_{-})|\sin (2{\theta }_{0})$ and the direction of $\overrightarrow{{r}_{BC}}$. In other words,

6 $$m\overrightarrow{{v}_{C}}({t}_{+})-m\overrightarrow{{v}_{C}}({t}_{-})=m|\overrightarrow{{v}_{C}}({t}_{-})|\sin (2{\theta }_{0})\overrightarrow{{r}_{BC}}/|\overrightarrow{{r}_{BC}}|.$$

By Newton’s third law, the model exerts an impulse equal in magnitude and opposite in direction on the ground or the treadmill belt, which is

7 $${\int }_{{t}_{-}}^{{t}_{+}}\overrightarrow{F}dt=-\,m|\overrightarrow{{v}_{C}}({t}_{-})|\sin (2{\theta }_{0})\overrightarrow{{r}_{BC}}/|\overrightarrow{{r}_{BC}}|.$$

Comparing the horizontal components of both sides of Eq. (7),

8 $${\int }_{{t}_{-}}^{{t}_{+}}{F}_{x}dt=m|\overrightarrow{{v}_{C}}({t}_{-})|\sin (2{\theta }_{0})\sin ({\theta }_{0}),$$

where *F*_*x*_ is the magnitude of the horizontal force exerted on the treadmill by the model. By Newton’s second law, this quantity directly contributes to the belt speed variation. For typical human walking, a speed increase induces an angle increase for the leading leg, *θ*_0_^18,19^. Therefore, Eq. (8) indicates that the impulse exerted on the treadmill belt and the resulting deceleration should be more prominent as the mass or the speed of the mass before HS increases.

**Figure 2:**
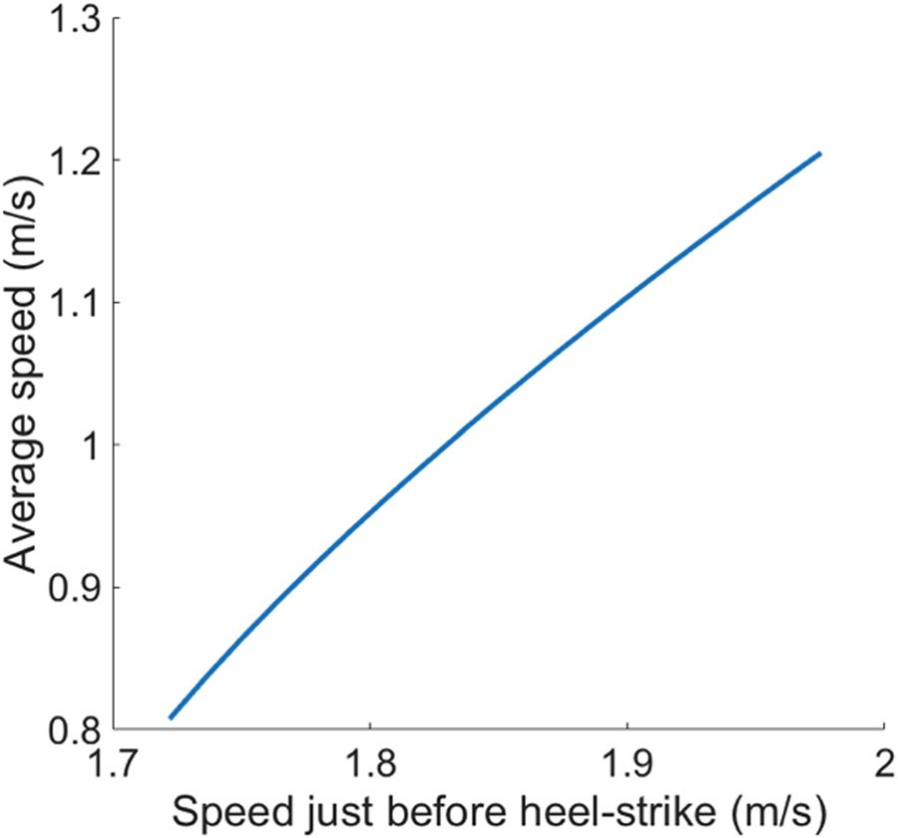
The average speed of the ankle actuated model versus the speed just before heel-strike (HS). The simple model exhibits asymptotically stable and periodic gait for a range of parameter values. In particular, the model shows cadence and average speed comparable to those of human walking when we assign humanlike parameter values to the model^14^. With given mass, leg length, foot length, and the angle of the leading leg (*θ*_0_), the average speed of the periodic gait of the model is determined by the actuation level. The ankle actuation constant *k* in Eq. (1) determines the speed. By controlling *k*, we changed the speed of the model just before HS and investigated the corresponding average speed. We let the model have the average speed range of 0.8–1.2 m/s, which is the speed range we investigated in the experiment. The maximum plantar ankle torque of the model, when it walks at 1.2 m/s, is close to the maximum ankle plantar torque of typical human walking reported by Perry^24^.

Though the speed of the mass before HS does not have to be exactly proportional to the average speed of the walker, which can be approximated as the controlled belt speed, we can reasonably expect a monotonic relation between the average locomotion speed and the speed of the mass before HS. The simulation result confirmed this prediction: the average speed of the model strongly, and almost linearly, depends on the speed before HS (Fig. 2). Accordingly, Eq. (8) implies that the impulse exerted on the treadmill belt increases as the mass or the average walking speed increases.

### A walking model with springy legs

**Figure 3:**
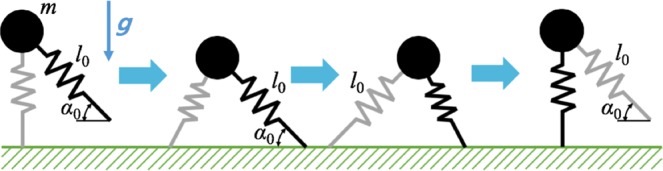
One step cycle of the springy legged walking model by Geyer *et al*.^16^. The first frame shows the model starting one step cycle at the apex of a single stance phase. The leading leg maintains a fixed angle of attack *α*_0_ until it touches the ground and begins the double stance phase (the second frame). During the double stance, the point mass keeps moving forward, and the trailing leg beings to extend; as soon as the length of the trailing leg recovers its rest length the double stance phase ends (the third frame). During the following single stance phase, the point mass reaches the apex, completing one step cycle.

The sequential configurations during one step of the model by Geyer *et al*. is shown in Fig. 3. The model starts at the apex of a single stance phase. The other swing leg remains in a fixed angle of attack *α*_0_ until it lands on the ground and initiates the double stance phase. As the point mass moves forward, the trailing leg reaches its rest length and terminates the double stance phase. During the following single stance phase, the point mass reaches the apex again, completing one step or half stride. This simple model successfully encapsulates the ground reaction force patterns of bipedal walking^16^.

The model can yield stable periodic gaits whose kinematics depend on the parameter values. With the constant rest length of the leg *l*_0_ and the angle of attack, the ground reaction force pattern of the stable periodic gait changes depending on the mass *m* and the non-dimensional stiffness $\tilde{k}=k{l}_{0}/mg$, where *k* is the stiffness of the springy legs. Though the model can yield asymmetric stable gaits as well, we confined our interest to symmetric gaits in which the ground reaction force pattern is similar to that of human walking.

**Figure 4:**
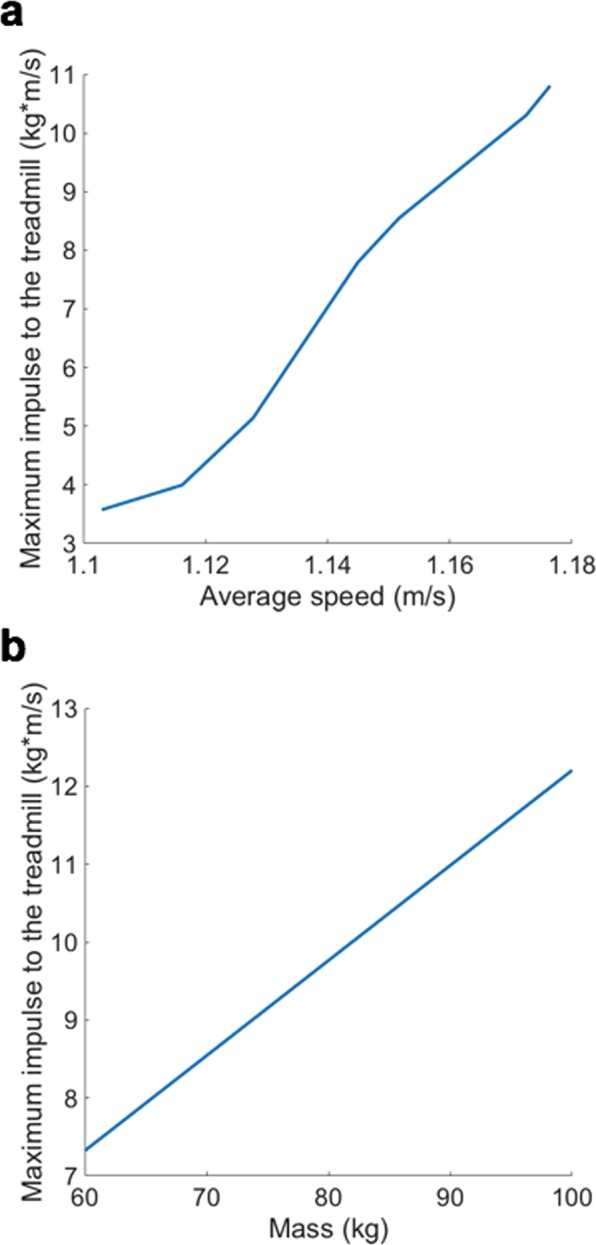
The impulse that the springy legged walking model by Geyer *et al*. exerts on the ground or the treadmill. Numerical simulation shows that the amount of impulse increase with the increases in the average speed of the model (**a**), and with the increase in the mass of the model (**b**).

Despite the simplicity of the model, analytical analysis of the model is challenging, and the amount of the horizontal component of the impulse that the model exerts on the ground or the treadmill belt needs to be obtained by numerical integration. By the linear momentum principle, the impulse, the time integration of the ground reaction force equals the changes in the linear momentum of the model;

9 $${\int }_{{t}_{1}}^{{t}_{2}}GR{F}_{x}dt=m\dot{x}({t}_{2})-m\dot{x}({t}_{1}),$$

where *t*_1_ and *t*_2_ are arbitrary time, *x* is the position of the point mass in horizontal direction, and *GRF*_*x*_ is the magnitude of the horizontal ground reaction force exerted on the model by the ground or the treadmill. By Newton’s 3^rd^ law, the impulse exerted on the ground or the treadmill has the opposite sign;

10 $${\int }_{{t}_{1}}^{{t}_{2}}{F}_{x}dt=m\dot{x}({t}_{1})-m\dot{x}({t}_{2}),$$

where *F*_*x*_ is the force that the model exerts on the treadmill. *F*_*x*_ is positive when the model decelerates as in the early phase of the double stance phase, and becomes negative when the model accelerates as in the terminal stance phase. The impulse keeps increasing as long as *F*_*x*_ is positive, and reaches its maximum when *F*_*x*_ becomes zero. The amount of impulse begins to decrease as soon as *F*_*x*_ becomes negative or the model begins to accelerate. Numerical simulations calculated the maximum impulse exerted on the ground or the treadmill by finding the maximum difference in the horizontal velocity. The non-dimensional stiffness $\tilde{k}$ changes the average speed of the periodic gait. We evaluated the maximum impulse across different values of $\tilde{k}$ and *m* to find how the impulse depends on the walking speed and the mass of the model. The springy legged model by Geyer *et al*. also predicts that the impulse exerted on the treadmill by the walker increases as the walking speed or the mass increases (Fig. 4).

To sum up, the mechanics of two simple models predicts that: 1) there exists a difference between the commanded treadmill speed and the actual belt speed, and the difference varies depending on the phase of the locomotion cycle, and 2) the amount of the error increases with increases in the weight of the walker, the average locomotion speed, or the commanded belt speed. We verified these predictions with experiments.

## Experimental Methods

### Subjects

Ten healthy, young men (age: 18–25, mean (SD): 21.4 (2.3); weight: 72.5–89.8 kg, 79.1 (5.3); height: 170.2–193.0 cm, 179.8 (8.7)) and ten healthy, young women (age: 18–23, 20.8 (2.1); weight: 50.8–77.3 kg, 62.8 (7.5); height: 154.9–172.7 cm, 163.8 (4.5)), with the ability to walk comfortably on a treadmill without assistance, participated in this study. All subjects were shown the functionality of the treadmill and its emergency shut-off capabilities. All aspects of this study conformed to the principles and guidelines described in the Declaration of Helsinki, and the Institutional Review Board of Arizona State University approved this study. Subjects provided informed, written consent prior to participation.

### Experimental setup and protocol

We performed all walking studies using an instrumented treadmill (Bertec Fully Instrumented Treadmill, Columbus, OH, USA). The treadmill consists of two separate belts, each with its own motor (MPL-A4540F, Allen Bradley, WI, USA) that permits the independent speed control of each belt. The treadmill is capable of precisely controlling the commanded speed (the maximum error was less than 0.4% of the commanded speed) without a human subject. The treadmill is also capable of measuring forces applied to each belt in three directions: anterior–posterior (AP), medial–lateral (ML), and vertical (i.e., superior–inferior). The maximum load range in the AP/ML and vertical directions is 2.5 kN and 5 kN, respectively. Since both sides of the treadmill have the same mechanical configuration and controller, we investigated the speed variation only in the left belt of the treadmill without losing generality. The treadmill was equipped with encompassing safety handles. Before recording data, subjects were familiarized with treadmill walking.

A motion capture system (VICON Bonita 10 System, UK) was used to directly measure the position of the belt and calculate its speed. Eight infrared cameras were used to track positions of passive reflective tape markers (in sets of three) attached along the side of the belt. The tape was thin enough (thickness: 0.015 cm; 3 M Scotchlite 7610 Reflective, MN, USA) not to interfere with belt movement. Three distinct triangles, in the shape of an equilateral, isosceles, and right triangle, were used to effectively calculate the speed of the belt throughout the entire gait cycle. Marker sets were positioned such that the cameras could capture at least one marker set at any instance of the gait cycle. Treadmill belt speed data, calculated by differentiating the position data, and ground reaction force data were filtered using a 4^th^ order Butterworth filter with cut-off frequencies of 10 Hz and 20 Hz, respectively. Ground reaction forces in AP, ML, and vertical directions were captured at 1 kHz, low-pass filtered, and down-sampled to match motion capture data, sampled at 100 Hz. Following a conventional notation, we defined AP, ML, and vertical directions as *x*, *y*, and *z*, respectively.

Each subject participated in three walking sessions with different treadmill speed settings: 0.8 (session 1), 1.0 (session 2), and 1.2 m/s (session 3). Each session lasted 5.5 minutes, and a minimum of 3-minute break was provided between sessions. Considering transient responses when speed changes from zero to the target speed, data from the first 0.5 minute of each session were not included in the data analysis.

### Data analysis

We estimated the instantaneous belt speed as time differentiation of the measured belt position. Based on the moment of HS, identified by the significant increase in the vertical ground reaction force (>10 N), velocity data was subdivided into multiple strides, and each stride interval was normalized in time to the 100% gait cycle. The velocity data, which changed depending on the normalized gait cycle, were then averaged to calculate the mean and standard deviation (SD) across strides. The mean and SD of the corresponding ground reaction force were calculated in the same way as the belt speed. Based on each subject’s averaged data sets, we calculate the following three quantities for each speed condition: 1) maximum speed changes around HS, 2) maximum speed changes around TO, and 3) the overall speed variation throughout the gait cycle. We also quantified the local maximum GRF_*x*_ around HS and TO for each speed condition to investigate the correlation with maximum speed changes around HS and TO.

To test our main hypothesis that both walking speed and subject weight have significant effects on treadmill speed changes, we performed a separate statistical analysis for each of the three dependent variables: the maximum speed change around HS, the maximum speed change around TO, and the overall speed variation throughout the gait cycle. We ran an analysis of covariance (ANCOVA) with walking speed as the independent variable and weight as the covariate or confounding variable. Following the ANCOVA, we performed post hoc comparisons with the Bonferroni correction. Further, we performed paired t-tests to investigate if there was any significant difference on the amount of treadmill speed changes between around HS and TO. We performed the same statistical analysis for maximum GRF_*x*_ around HS and TO, which helped the interpretation of the statistical analysis on treadmill speed changes. In all statistical analyses, we checked the normality of data by running Shapiro–Wilk tests and evaluated equal variance (homogeneity of variance) across data sets by running Levene’s tests. If the null hypothesis was rejected in the Levene’s test, equal variance was not assumed in the subsequent statistical analyses. All statistical tests were made using the SPSS statistical package at a significance level of *p* < 0.05.

We also calculated the Pearson correlation coefficient (*r*) between treadmill speed changes and the corresponding GRF_*x*_. Finally, we performed a multiple linear-regression analysis to test whether walking speed and subject weight could account for treadmill speed change using the following model: maximum speed variation = intercept + c_weight_ × weight + c_walking speed_ × walking speed, where the c_weight_ and c_walking speed_ are the partial coefficients of regression.

In addition to the statistical analysis, all measured and analyzed data were group-averaged and plotted for the following six different conditions: three speed conditions (0.8, 1.0, and 1.2 m/s) and two weight conditions (top 50% and bottom 50% weight group). We divided the data into two weight groups in order to clearly visualize the effect of subject weight on treadmill speed changes and ground reaction forces.

## Results

### Walking speed and subject weight had significant effects on treadmill speed changes

**Figure 5:**
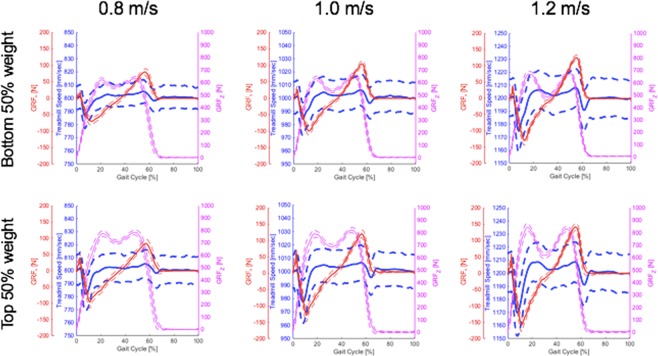
Treadmill speed and ground reaction force in the anterior–posterior direction change depending on gait cycle. Blue: Treadmill speed, Red: Ground reaction force in the anterior–posterior direction, Magenta: Ground reaction force in the vertical direction. Solid and dotted curves denote the mean and the mean ± 1 SD, respectively.

Treadmill speed was not constant throughout the gait cycle, and both walking speed and subject weight had significant influence on treadmill speed changes. In particular, the speed substantially changed around HS and TO, but the amount of speed change was greater around HS than TO (Fig. 5). The difference of the amount from HS to TO was 14.5, 16.1, and 18.4 mm/s for the treadmill speed settings of 0.8, 1.0, and 1.2 m/s, respectively. Paired t-tests confirmed that these differences are statistically significant (*p* < 0.001).

**Figure 6:**
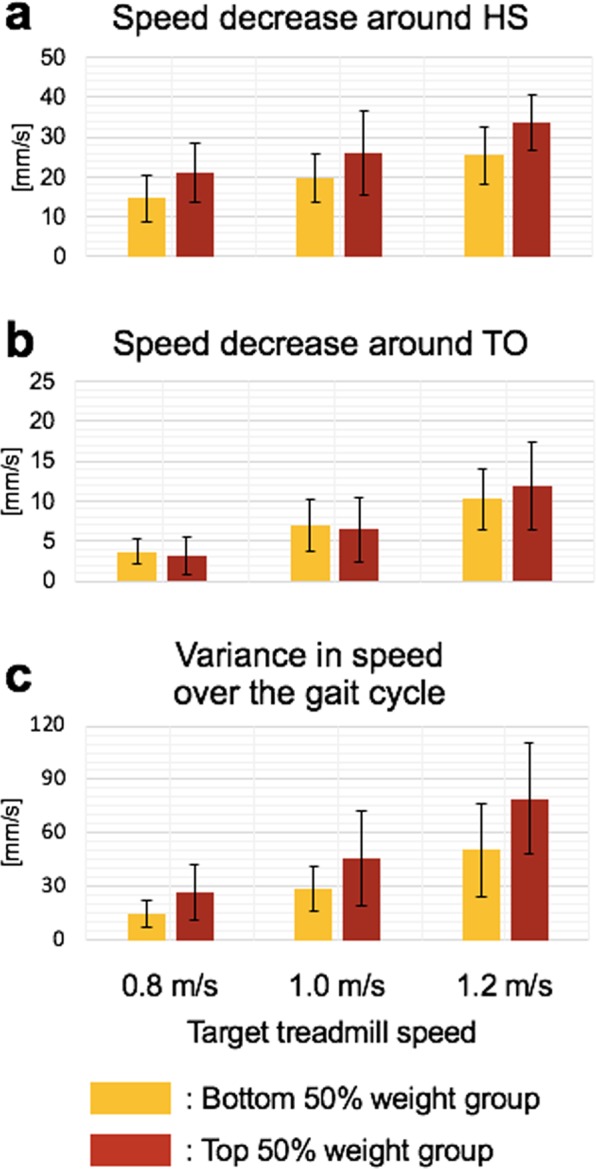
Effects of walking speed and subject weight on treadmill speed changes. Bars and error bars denote the mean and the mean ± 1 SD, respectively. Yellow and red bars represent results of the bottom 50% weight group and the top 50% weight group, respectively.

The amount of treadmill speed change around HS increased with increasing walking speed and subject weight (Fig. 6a). Statistical analysis showed that there was a significant effect of walking speed on the treadmill speed change around HS after controlling for the effect of weight, *F*(2, 56) = 12.02, *p* < 0.001. Post hoc comparisons also revealed that the amount of speed change increased with increasing walking speed (Table 1). In addition, the covariate, weight, was significantly related to the speed change around HS, *F*(1, 56) = 10.48, *p* = 0.002.

**Table 1:** Post hoc comparisons for the amount of treadmill speed change.

Gait Instance	Groups to Be Compared	Mean Difference [mm/s]	Significance (*p*)
Heel-Strike (HS)	1.2 m/s–1.0 m/s	6.65	0.021
	1.2 m/s–0.8 m/s	11.60	<0.001
	1.0 m/s–0.8 m/s	4.95	0.125
Toe-Off (TO)	1.2 m/s–1.0 m/s	4.38	0.001
	1.2 m/s–0.8 m/s	7.68	<0.001
	1.0 m/s–0.8 m/s	3.30	0.016

The amount of treadmill speed change around TO also increased with increasing walking speed, but the weight effect was not statistically significant (Fig. 6b). There was a significant effect of walking speed on the treadmill speed change around TO after controlling for the effect of weight, *F*(2, 56) = 23.10, *p* < 0.001. Post hoc comparisons also revealed that the amount of speed change increased with increasing walking speed (Table 1). However, weight was not significantly related to the speed change around TO, *F*(1, 56) = 0.23, *p* = 0.637.

The treadmill speed variation throughout the gait cycle increased with increasing walking speed and subject weight (Fig. 6c). A significant effect of walking speed on the speed variation was identified after controlling for the effect of weight, *F*(2, 56) = 20.79, *p* < 0.001. In addition, weight was significantly related to the speed variation, *F*(1, 56) = 9.37, *p* = 0.003.

### Treadmill speed changes were due to the force from the foot to the belt

As expected from the linear momentum principle, both walking speed and subject weight had significant influence on GRF_*x*_, which was highly correlated with prominent changes in treadmill speed. The local maximum of GRF_*x*_ occurred around HS and TO (Fig. 5).

**Figure 7:**
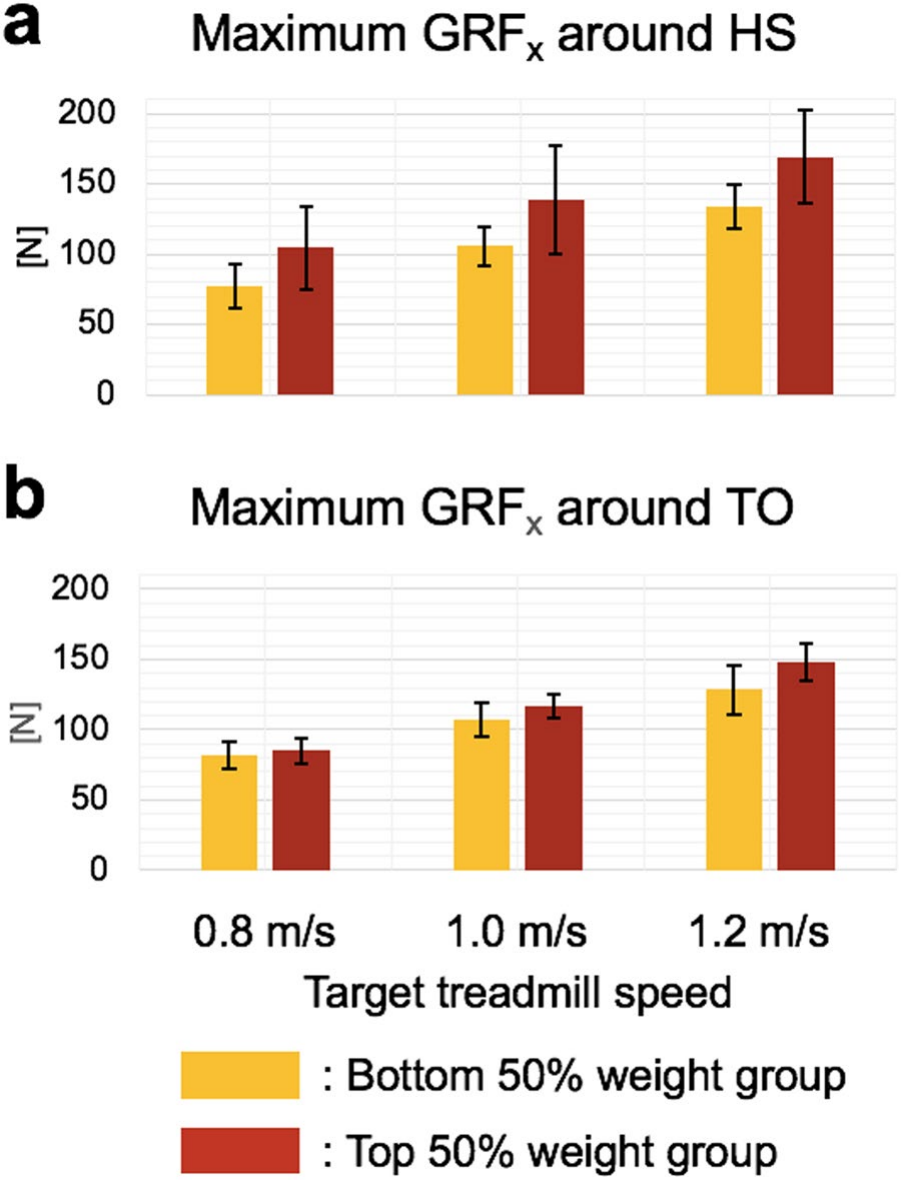
Effects of walking speed and subject weight on ground reaction force. Bars and error bars denote the mean and the mean ± 1 SD, respectively. Yellow and red bars represent results of the bottom 50% weight group and the top 50% weight group, respectively.

The local maximum GRF_*x*_ around HS increased with increasing walking speed and subject weight (Fig. 7a). Statistical analysis showed that there was a significant effect of walking speed on the local maximum GRF_*x*_ around HS after controlling for the effect of weight, *F*(2, 56) = 25.18, *p* < 0.001. Post hoc comparisons also revealed that the local maximum GRF_*x*_ around HS increased with increasing walking speed (Table 2). In addition, the covariate, weight, was significantly related to the local maximum GRF_*x*_ around HS, *F*(1, 56) = 17.30, *p* < 0.001.

**Table 2:** Post hoc comparisons for the local maximum GRF_x._

Gait Instance	Groups to Be Compared	Mean Difference [N]	Significance (*p*)
Heel-Strike (HS)	1.2 m/s–1.0 m/s	29.19	<0.001
	1.2 m/s–0.8 m/s	60.51	<0.001
	1.0 m/s–0.8 m/s	31.32	<0.001
Toe-Off (TO)	1.2 m/s–1.0 m/s	26.31	<0.001
	1.2 m/s–0.8 m/s	55.03	<0.001
	1.0 m/s–0.8 m/s	28.72	<0.001

The local maximum GRF_*x*_ around TO also increased with increasing walking speed and subject weight (Fig. 7b). There was a significant effect of walking speed on the local maximum GRF_*x*_ around TO after controlling for the effect of weight, *F*(2, 56) = 134.95, *p* < 0.001. Post hoc comparisons also revealed that the local maximum GRF_*x*_ around TO increased with increasing walking speed (Table 2). In addition, weight was significantly related to the local maximum GRF_*x*_ around TO, *F*(1, 56) = 37.49, *p* < 0.001.

**Figure 8:**
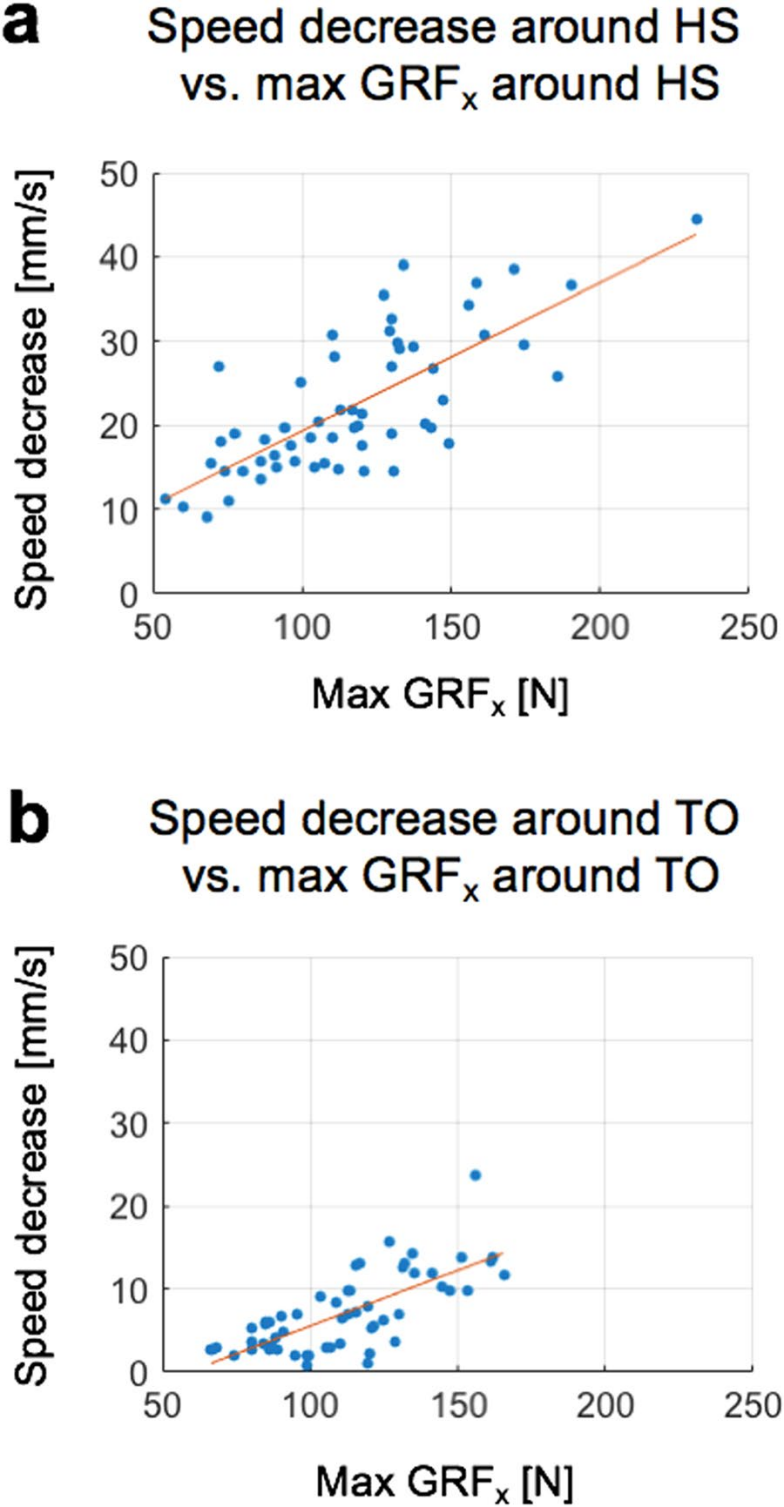
Treadmill speed decrease and ground reaction force are highly correlated. Blue dots: measurements, Red line: linear fitting.

Significant speed changes around HS and TO were highly correlated with the corresponding local maximum of the amplitude of GRF_*x*_ (Fig. 8). The Pearson correlation coefficients between the two variables were *r* = 0.75 (*p* < 0.001) and *r* = 0.73 (*p* < 0.001) around HS and TO, respectively. The R^2^ of the multiple linear-regression analysis around HS was 0.46, and the coefficients in regression analysis were statistically significant (*p* = 0.002 and *p* < 0.001 for c_weight_ and c_walking speed_, respectively). The R^2^ of the multiple linear-regression analysis around TO was 0.47. The coefficient of regression for walking speed (c_walking speed_) was statistically significant (*p* < 0.001) whereas the one for weight (c_weight_) was not (*p* = 0.707).

## Discussion

The difference between treadmill and overground locomotion has been widely reported. Many studies have suggested psychological causes of the difference, including visual information and fear. It is certainly plausible that these psychological effects contribute to the different motor output like kinematics, kinetics, muscle activation, and stability. However, unlike the quantifiable motor output, the suggested psychological causes of the difference can hardly be quantified. Consequently, the actual effect of proposed psychological causes has not yet been systematically addressed. In this study, we attended to causes that can be quantified and controlled, i.e. the mechanical difference between treadmill and overground locomotion.

The experimental results are consistent with the prediction based on mechanics. In particular, contrary to the result of a previous study^12^, we demonstrated that the speed of locomotion significantly contributes to the belt speed error. Locomotion is accompanied by a patterned ground reaction force, and the reaction force from a foot to the treadmill belt—which depends on the speed as well as the weight—should inevitably affect the dynamics of the treadmill. In fact, statistical analyses showed that the effect of walking speed on the belt speed error was more prominent than the effect of weight. This is consistent with a prediction from the highly simplified walking model with ankle actuation. Though the model extensively, but deliberately, omits physiological and anatomical realism, it successfully informs us that the amount of horizontal impulse exerted on the treadmill belt depends on the mass, the speed of the center of mass before HS, and the angle of the leading leg, *θ*_0_. We did not estimate the leg angle of the subjects, so we cannot show the actual angle of the leading leg from our experimental data. However, it is known that humans increase stride length to walk faster^18,19^. Consequently, the angle of the leading leg (*θ*_0_) should increase with locomotion speed. In addition, the relation between the speed before HS and the average speed is almost linear (Fig. 2). Therefore, considering Eq. (8), the model predicts that the effect of average speed on the horizontal component of the impulse is larger than the effect of mass.

Although the horizontal force profile during stance phase possesses approximate point symmetry, the profile of the treadmill belt speed does not (Fig. 5). The non-ideal behavior of the belt speed was clearly more evident around the HS phase than around the TO phase. We speculate that a few factors induced these results. First, the increase of the decelerating force during the loading of the leading leg was much faster than the increase of the force during the stance phase. Considering the finite sampling rate of the feedback loop, the rapidly developed decelerating force during the HS phase can affect the belt speed even before the speed controller tries to compensate for the speed error. In contrast, the force during the stance phase develops relatively slowly, allowing the treadmill system enough time to control the belt speed. It is also probable that the belt slips over the rotating drum due to the loaded external force. In particular, a large and rapidly increasing braking force is exerted during the landing of a foot. A slip between the belt and the drum may contribute to the largest amount of the speed change around HS.

This study alone cannot address whether and how much the observed treadmill belt speed error is responsible for the reported difference between treadmill and overground walking in kinematics, kinetics, and muscle activation patterns. However, the previous study by Savelberg *et al*. demonstrated a significant correlation between the treadmill belt speed error and the kinematic differences between treadmill and overground locomotion^12^. Although the exact physiological or biomechanical mechanism how the treadmill belt speed error affects human walking has not been revealed, the significant correlation between the belt speed error and the quantified differences between treadmill and overground locomotion strongly supports that the belt speed error is at least partly responsible for the observed difference between treadmill and overground locomotion. This finding, combined with the results of the current study, suggests that the difference between treadmill and overground walking will be amplified by the increase in the walking speed and the weight of the walker.

A previous study with a similar experimental setup estimated the mechanical energy exchange between a subject and a treadmill by measuring the belt speed deviation and the ground reaction force^13^. The study concluded that, although the deviation of the belt speed is over 3%, the total energy exchange is less than 1.6% of the work performed on the center of mass, so treadmill walking is only mildly disturbed by the non-ideal mechanical behavior of the treadmill. Our results also show that the average difference between the maximum and minimum belt speed is 3.1% of the commanded belt speed at the walking speed of 1.2 m/s. We agree that the mechanical work done on the center of mass due to the belt speed error can be small, but we speculate that the small amount of energy exchange does not always guarantee negligible disturbance. Motor neuroscience studies emphasize the critical role of cutaneous sensory input through the foot in the regulation of human locomotion^19–21^. If the sensory input from the foot is critical in locomotor control, the belt speed difference of up to 3% may affect the locomotor output significantly, and the resulting effect may contribute to the noticeable difference between treadmill and overground locomotion. A study by Roll *et al*. actually demonstrated that a change in cutaneous afferents from the plantar sole significantly alters the path of center of pressure during locomotion^22^. Nurse *et al*. also showed that supra-sensory vibration applied to the sole changes the location of center of pressure^23^. It is necessary to note that the mechanical energy exchange due to such foot sensation is negligible, whereas the effect of the sensation on gait and posture is significant.

Furthermore, the error up to 3% is not randomly assigned: the treadmill belt speed changes periodically depending on the gait phase (Fig. 5). Therefore, treadmill locomotion fundamentally requires motor adaptation to a dynamic environment, which is *mechanically* different from the stationary ground. Suppose that we walk on the ground, and the ground moves toward our center of mass with a speed of 3% of our intended walking speed at every heel-strike. It is plausible that our neuro-motor system will adapt to the novel environment and use a new motor control strategy, resulting in different kinematics and muscle activation patterns.

The current study investigated only a limited range of walking speeds. Each subject walked at 0.8, 1.0, and 1.2 m/s. Our analyses showed clear dependence of the treadmill speed error on the average speed even within this narrow range of walking speeds. We expect that the R^2^ value for the multiple regression would increase if we investigated the effect of a wider range of walking speeds. Another limitation is a potential systematic effect due to the non-randomized study design regarding the walking speed sequence. Although subjects were sufficiently familiarized with treadmill walking before the main study, any adaptation behavior in treadmill walking could not be controlled in the sequential study design.

In this study, we used a split-belt treadmill, which allows one foot per belt. When we walk on a typical single belt treadmill, both feet exert force to the belt during the double stance phase, so the resultant belt speed error is expected to be different. This limitation is inevitable as long as we are to obtain the exact force data from each foot and analyze the effect of ground reaction force on the belt speed error. A previous study, which used single belt treadmills without directly assessing the reaction force, reported that the belt speed variation was 3% for a high power treadmill designed for training horses and 6% for a typical treadmill designed for routine clinical gait analysis and rehabilitation^12^. According to the result of this previous study, the speed variation of a typical single belt treadmill is not less than what we observed from the instrumented split-belt treadmill.

## Conclusion

We periodically exert force on the ground when we walk or run. If a slip between the treadmill belt and the rotating drum occurs due to the external force, the treadmill belt already fails to serve as an inertial frame of reference. Even if we assume no slip between the belt and the rotating drum, the force from a foot to the belt provides significant perturbation to the drum and the motor, and the motor of a treadmill is not an ideal flow source. Due to the limited power of the electrical motor and the finite sampling rate of the controller, the treadmill belt speed cannot be constant when someone walks or runs on the treadmill.

The linear momentum principle predicts that the speed and the weight of the walker should affect the treadmill speed error, and our experiment confirmed this prediction. Though many studies have reported the difference between treadmill and overground locomotion, either the experiment design criterion for a treadmill locomotion study or the system requirement of a treadmill platform that replicates overground locomotion has not been addressed systematically. We perceive that such design criteria cannot be developed if we attribute the difference between treadmill and overground locomotion only to psychological effects, which can hardly be quantified. To initiate the first step in the development of the necessary criteria, our study addressed the effect of quantifiable causes (speed and weight) on the quantifiable non-ideal behavior of a treadmill (belt speed error). A previous study demonstrated that the intra-stride variation of the treadmill belt speed depends on the power of the electrical motor of the treadmill^12^. In a feedback control system, the sampling frequency of the controller, or the loop time inevitably affects the error of the controlled variable, which is the belt speed in the case of treadmill. Therefore, at least design criteria for the power of the motor and the speed of the controller need to be developed by additional future work. A competent mathematical model of a treadmill system and systematic experimental studies with various treadmill specifications will contribute to establishing concrete design criteria for a treadmill platform, which specifies the requirements for motor power and controller speed when the acceptable belt speed error, required walking speed range, and weight range of the participating walkers are specified.

## Data Availability

The data sets generated during and/or analyzed during the current study are available from the corresponding author on reasonable request.
